# Modelling Caffeine and Paracetamol Removal from Synthetic Wastewater Using Nanofiltration Membranes: A Comparative Study of Artificial Neural Networks and Response Surface Methodology

**DOI:** 10.3390/membranes15080222

**Published:** 2025-07-24

**Authors:** Nkechi Ezeogu, Petr Mikulášek, Chijioke Elijah Onu, Obinna Anike, Jiří Cuhorka

**Affiliations:** 1 Institute of Environmental and Chemical Engineering, Faculty of Chemical Technology, University of Pardubice, Studentská 573, 532 10 Pardubice, Czech Republic; nkechi.ezeogu@student.upce.cz (N.E.); obinna.anike@student.upce.cz (O.A.); jiri.cuhorka@upce.cz (J.C.); 2 Department of Chemical Engineering, Nnamdi Azikiwe University, Awka 5025, Nigeria; 3 Institute of Metallurgy, Clausthal University of Technology, Robert-Koch-Straße 42, 38678 Clausthal-Zellerfeld, Germany

**Keywords:** caffeine, paracetamol, artificial neural networks (ANN), response surface methodology (RSM), nanofiltration (NF)

## Abstract

The integration of computational intelligence techniques into pharmaceutical wastewater treatment offers promising opportunities to improve process efficiency and minimize operational costs. This study compares the predictive capabilities of Response Surface Methodology (RSM) and Artificial Neural Network (ANN) models in forecasting the rejection efficiencies of caffeine and paracetamol using AFC 40 and AFC 80 nanofiltration (NF) membranes. Experiments were conducted under varying operating conditions, including transmembrane pressure, feed concentration, and flow rate. The predictive performance of both models was evaluated using statistical metrics such as the Coefficient of Determination (R^2^), Root Mean Square Error (RMSE), Marquardt’s Percentage Squared Error Deviation (MPSED), Hybrid fractional error function (HYBRID), and Average Absolute Deviation (AAD). Both models demonstrated strong predictive accuracy, with R^2^ values of 0.9867 and 0.9832 for RSM and ANN, respectively, in AFC 40 membranes, and 0.9769 and 0.9922 in AFC 80 membranes. While both approaches closely matched the experimental results, the ANN model consistently yielded lower error values and higher R^2^ values, indicating superior predictive performance. These findings support the application of ANNs as a robust modelling tool in optimizing NF membrane processes for pharmaceutical removal.

## 1. Introduction

The world’s rivers are full of pharmaceuticals, which is unsustainable. Wilkinson et al. [1] obtained samples from 258 rivers across all continents. They analysed each sample for traces of pharmaceuticals. Their findings showed that only one indigenous village in Venezuela, where modern medicines are not used, and Iceland were free from contamination. In all other rivers, traces of pharmaceuticals were detected. The findings of their study are conclusive and provide strong evidence for the presence of pharmaceutically active compounds (PhACs) in water bodies, which potentially threaten aquatic ecosystems and human health. These PhACs in marine ecosystems originate from domestic sewage, hospital effluent, pharmaceutical manufacturing industries, animal husbandry, and other sources [2].

To address these issues, pharmaceutical wastewater typically undergoes a series of treatment processes. The primary treatment focuses on removing suspended solids and particulate matter, while the secondary treatment targets the reduction of biodegradable organic compounds. Despite the effectiveness of these stages, trace amounts of suspended solids and dissolved organic matter may still exist in the wastewater [3]. These residual contaminants can pose significant environmental risks if discharged without adequate treatment. As a result, the continued development and improvement of advanced wastewater treatment technologies are essential to ensure the safe and effective treatment of pharmaceutical and other industrial wastewater.

Pressure-driven membrane processes are the most widely used among the various methods used for advanced wastewater treatment. These processes operate on the principle of hydraulic pressure to effect separation. The four main types of pressure-driven membrane processes are microfiltration (MF), ultrafiltration (UF), nanofiltration (NF), and reverse osmosis (RO) [4]. Compared to other pressure-driven membrane processes, NF is well known for its efficiency in separating small particles with a pore size between 0.1 and 1 nm; NF membranes allow water and monovalent salts to pass through the membrane while retaining multivalent ions, low-molecular-weight molecules, sugars, proteins, and other organic compounds. This technology has become a promising solution for removing pharmaceuticals from contaminated wastewater, combining high selectivity, energy efficiency, and cost-effectiveness. The NF membrane is influenced by several factors, including the chemical composition of the feed solution (water characteristics, pH, ionic strength, and solute concentration) and separation conditions (transmembrane pressure, feed flow rate, and temperature) [5]. Additionally, the membrane’s properties, including its hydrophilicity or hydrophobicity, surface charge, morphology, pore size, and roughness, play significant roles in the membrane fouling [5]. These parameters have been extensively studied in various research studies [6,7].

Pharmaceutical contaminants, including analgesics and stimulants, are among the most frequently detected pollutants in rivers across many countries [1]. Paracetamol (acetaminophen), a widely used analgesic, is one of the most common micropollutants in the environment due to its high water solubility, presence in more than 600 over-the-counter medicines [8], and low biodegradability [9]. Similarly, caffeine, a common stimulant and lifestyle pharmaceutical compound, is prevalent in aquatic environments due to its widespread consumption and subsequent discharge. Given their universality, environmental persistence, and potential ecological impact, paracetamol and caffeine were selected as model pollutants for this study to investigate their removal efficiency in an NF process.

In membrane science and technology, modelling has emerged as a valuable tool for predicting membrane separation processes by establishing a relationship between system variables and their responses. This capability not only aids in understanding complex interactions within membrane systems but also offers practical advantages. Using numerical mathematical methods, modelling helps save costs and time by reducing the need for extensive physical experimentation. Furthermore, it enhances process efficiency, enables predictive modelling, minimizes errors, and optimizes resource use. These benefits make it a critical tool for researchers and industries seeking to streamline chemical process development and operations [10].

Some modelling tools that can solve linear and nonlinear multivariate regression problems are Response Surface Methodology (RSM) and Artificial Neural Networks (ANNs).

In recent years, RSM based on the design of experiments (DoE) has been applied successfully in different areas of membrane technology [5,11,12,13,14,15,16]. RSM is a widely used statistical and mathematical tool for experimental design and optimization, particularly in complex systems involving multiple input parameters and searching optimum conditions of variables to predict targeted responses [17]. The input parameters that affect the process are called independent variables, while the responses are called dependent variables [18].

RSM provides a structured framework with three key components: experimental design, model fitting, and optimization [19]. The experimental design phase ensures accuracy by spanning a broad range of input variable values, using designs such as the Central Composite Design (CCD), the Box–Behnken design (BBD), the Central Composite Rotatable Design (CCRD) [18], or a custom design [20]. Subsequently, a mathematical model is developed to describe the relationship between inputs and the response, typically expressed as a polynomial equation. Regression methods, such as least squares, estimate the model’s coefficients. Finally, the optimization phase identifies the ideal input values that yield the desired response.

Similarly, RSM has proven to be a valuable tool for optimizing various membrane-based processes. For instance, Mohammad et al. [21] applied RSM based on BBD to determine the optimal conditions for fabricating polyethersulfone (PES) NF membranes by phase inversion. Four essential parameters, including PES polymer concentration, polyvinylpyrrolidone (PVP) concentration, evaporation time, and coagulation bath temperature, were the factors influencing the separation process, and the optimization objectives were water flux and rejection. The analysis of variance shows that all four independent parameters are statistically significant, and the final model is reasonably accurate. This research underscores the adaptability of RSM in optimizing operational parameters and improving process efficiency across various membrane technology applications.

On the other hand, an Artificial Neural Network (ANN) is a computational model inspired by the superior qualities of the human brain, designed to recognize patterns and solve complex problems. This modern computational approach utilizes nonlinear modelling and complex datasets [22]. The system is designed to operate as a black box model that describes the relationship between the input and the output system [23] without needing to understand the physical characteristics of the process [24]. A typical ANN consists of an input (independent) layer, one or more hidden layers with many hidden neurons, and an output (dependent) layer [25]. Weights and biases mathematically link these layers [26]. This relationship is established from a learning process in which a dataset is provided to the network; hence, it is a data-driven model.

Coskun et al. [27] developed an ANN model to analyse flux behaviour during cross-flow microfiltration of a phosphate and fly ash wastewater mixture under varying conditions. Two ANN architectures (NN1, NN2) were designed and evaluated using different algorithms and network types. The models demonstrated superior predictive accuracy, achieving high correlation values (R^2^ = 0.991 and 0.988), low error distributions, and precise normalized flux predictions. This highlights ANNs’ capability to reliably capture the effects of key process variables, such as time, pressure, concentration, and membrane type, on flux behaviour.

In parallel with traditional modelling strategies like ANNs and RSM, recent studies have explored the integration of bio-inspired metaheuristic algorithms, such as Particle Swarm Optimization and Grey Wolf Optimization, into classical membrane transport models like the Spiegler–Kedem equation [28]. Although not within the scope of this study, these approaches offer promising avenues for enhancing prediction accuracy and process robustness in future membrane separation research.

Recent advances in membrane technology have also focused on enhancing treatment performance by integrating structural innovation with functional mechanisms. For example, hierarchically structured hybrid membranes that couple adsorption with membrane ultrafiltration have been proposed for continuous wastewater treatment, offering improved pollutant retention and operational stability [29]. In another study, a multiscale 3D wet-electrospun “branch leaf” architecture of graphene oxide–polycaprolactone fibres demonstrated excellent oil–water separation performance through optimized structural design [30]. While these works emphasize material and structural advances, our approach complements this trend by focusing on process-level optimization and predictive modelling for pharmaceutical rejection in nanofiltration systems.

This study aims to evaluate and compare the performance of two statistical mathematical models, RSM and an ANN, to predict and optimize pharmaceutical rejection using polymeric composite membranes (AFC 40 and AFC 80) in NF processes. Experimental data were used to model and predict the rejection behaviour of two pharmaceutical compounds: caffeine and paracetamol. The analysis considered key input variables such as feed concentration (5, 10, and 20 mg/L), transmembrane pressure (10, 15, 20, 25, 30 bar), and feed flow rate (5, 10, and 15 L/min) to forecast pharmaceutical rejection efficiencies. Caffeine rejection was assessed using AFC 40 membranes, while paracetamol rejection was evaluated using AFC 80 membranes. Notably, this work presents the first direct comparison of RSM and an ANN in the context of caffeine and paracetamol removal through NF membranes, contributing valuable insights into model accuracy and process optimization in pharmaceutical wastewater treatment.

## 2. Materials and Methods

### 2.1. Feed Solution Preparation

The pharmaceuticals selected for this study have molecular weights of 194.2 and 151.2 g/mol (caffeine and paracetamol), respectively. To carry out this experiment, each of these pharmaceuticals was diluted in pure water at 300 mg/L, and these stock solutions were stored in a refrigerator at approximately 5 °C.

The pharmaceutical tablets were ground using a mortar and pestle to prepare the feed solution, followed by ultrasonication of the solution for 30 min. This solution was then filtered using a 0.7 µm glass filter in a dead-end filtration unit at a 0.5 bar vacuum and diluted as required. All solutions were prepared using distilled water with a conductivity of approximately 15 μS/cm.

### 2.2. Experimental Set-Up and Procedures

The membranes were received as tubular polymeric NF membranes (AFC 40 and AFC 80). They were purchased from PCI Membrane Systems, Kostrzyn, Poland. They were gently washed with demineralized water to remove preservatives and stored in demineralized water at 25 °C. The membranes were first wetted in demineralized water for at least 24 h. Before the NF experiments, the membranes were compacted under 31 bar transmembrane pressure with demineralized water as the feed for 2 h until there was no variation in permeate flux.

The NF unit consisted of a 10 L feed tank equipped with an NF membrane module (FT 18, Armfield, GB, Ringwood, UK). The membrane module contained two tubular AFC 40 membranes for separating solutes from wastewater. Furthermore, caffeine was removed from the pharmaceutical-contaminated water using two tubular AFC 80 membranes. The pharmaceutical separation was carried out individually, and the conditions were optimized.

**Figure 1:**
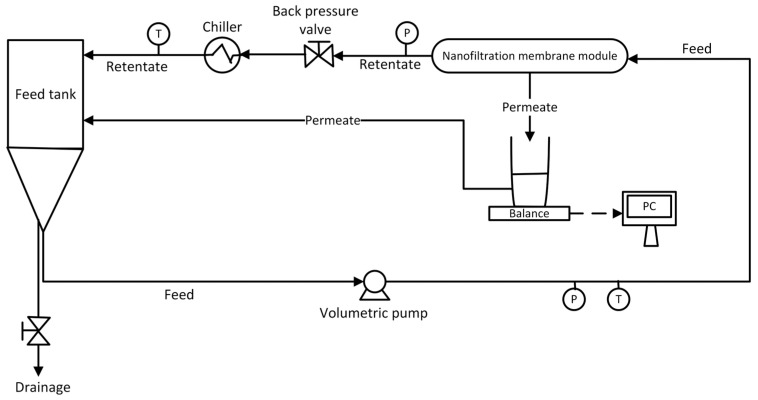
Schematic diagram of experimental NF system.

A pH meter (inoLab pH 7110-pH/mV meter + SenTix 21 electrode, Xylem Analytics GmbH, Weilheim, Germany) was incorporated into the feed tank, and a pH ranging from 6.3 to 6.6 was maintained. The feed solution temperature was maintained at 25 ± 0.5 °C using a heat exchanger (TAEevo, Armfield, GB, Ringwood, UK). The NF experiments operated in a total recirculation mode in which concentrate and permeate streams were returned to the feed tank to keep the feed concentration constant. Finally, a weighing machine (Balance KERN KB, Balingen, Germany) with an accuracy of ±0.01 g gravimetrically measured the permeate flux. A schematic diagram of the NF unit is shown in Figure 1.

### 2.3. Pharmaceutical Rejection Tests

Pharmaceutical rejections were evaluated using 5, 10, and 20 mg/L of caffeine and paracetamol solutions. The transmembrane pressure for the AFC 40 and AFC 80 membranes was varied in the range of 10–30 bar. The feed solution pH ranged from 6.3 to 6.6, while the feed solution temperature was kept constant at 25.0 ± 0.5 °C throughout the experiments. Feed and permeate samples were collected after the system was equilibrated for 1 h under each condition and analysed as described in Equations (1) and (2). The solute rejection performance ($R_{obs},\%$) was calculated based on the concentration of each PhAC in the permeate ($C_{p}$, mg/L) and feed ($C_{f}$, mg/L) streams, as shown in Equation (1).

(1) $$R_{obs}=\left(1-\frac{C_{p}}{C_{f}}\right)\times 100\%$$

The pharmaceutical concentration in the feed solution ($C_{f}$) and permeate ($C_{p}$) were calculated by using high-performance liquid chromatography (HPLC, Agilent 1260 infinity II Prime LC, Santa Clara, CA, USA). A sample volume of 1 μL was injected into a C18 column (5 μm, 250 mm × 4 mm) and a diode array detector (DAD). A mobile phase gradient of a solvent comprising 45% acetonitrile and 55% water at a flow rate of 0.5 mL min^–1^ was used.

The permeate flux ($J_{p}$) was determined by measuring the volume of permeate (ΔV, L) collected at regular time intervals (Δt, h), as follows:

(2) $$J_{p}=\left(\frac{\Delta V}{A\times \Delta t}\right)$$

where A is the membrane cell’s effective area (m^2^).

### 2.4. Experimental Design

#### 2.4.1. Modelling of NF Membranes Using RSM

A custom design approach involving RSM was employed to evaluate and optimize the operational parameters influencing pharmaceutical rejection by NF membranes. The modelling and experimental design were performed using Design-Expert^®^ software (Version 22.0, Stat-Ease Inc., Minneapolis, MN, USA). This approach allowed flexibility in defining specific experimental runs tailored to the research objectives, rather than relying on standard designs such as Box–Behnken or Central Composite Design.

Three independent variables were considered: pressure (X_1_), feed concentration (X_2_), and feed flow rate (X_3_). Following previous methodologies [31,32], the experimental rejection efficiency served as the response input for the polynomial model as described in Equation (3). Their ranges and levels are mentioned in Table 1.

(3) $$Y=\beta_{o}+\sum_{j=1}^{k}\beta_{j}x_{j}+\sum \sum_{i<j}\beta_{ij}x_{i}x_{j}+\sum_{j=1}^{k}\beta_{jj}x_{j}^{2}+\varepsilon$$

where Y is the predicted response variable; $\beta_{o},\;\beta_{j},\;\beta_{ij}$, and $\beta_{jj}$ are constant coefficients; *k* is the number of factors studied and optimized in the experiment; $x_{i}$ and $x_{j}$ are the coded independent variables or factors; and ε is the random error. The average values of duplicated experimental runs were calculated to ensure precision.

The custom RSM design generated a quadratic polynomial model incorporating linear, interaction, and squared terms. The experimental data were fitted to this model to derive a predictive equation capable of simulating system behaviour under varying conditions. The adequacy of the model was assessed using analysis of variance (ANOVA), the Coefficient of Determination (R^2^), and residual diagnostics. Finally, the optimized operating conditions for maximum pharmaceutical rejection were determined by solving the polynomial equation and analysing response surface plots.

**Figure 2:**
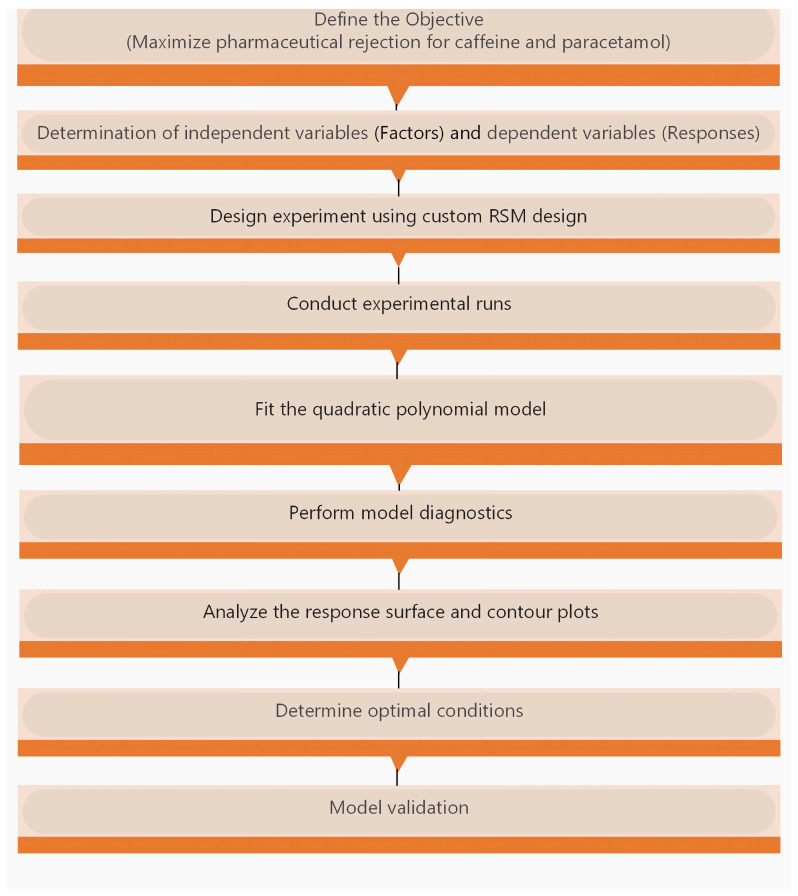
Steps for Response Surface Methodology.

The model calculates the coefficients for each main effect, interaction effect, and quadratic effect. Optimal conditions were determined by solving the quadratic equation and analysing the resulting response surface and contour plots. The key steps for implementing RSM are summarized in Figure 2.

#### 2.4.2. Modelling of NF Membranes Using ANN

**Figure 3:**
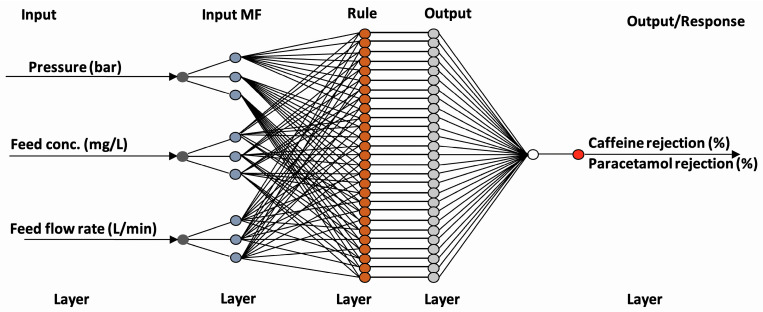
ANN architecture of pharmaceutical rejection process.

ANN modelling was employed to predict the rejection of pharmaceuticals using nanofiltration membranes. The analysis was conducted using the Neural Network Toolbox of MATLAB 7.0.1 (The MathWorks Inc., Natick, MA, USA). A multilayer perceptron (MLP) architecture was adopted for the modelling framework. Figure 3 shows the ANN architecture of the pharmaceutical rejection process.

The input variables for the ANN model were pressure, feed concentration, and feed flow rate, identical to those used in the RSM analysis. The output variable was the pharmaceutical rejection percentage. A supervised learning approach was used, applying the Levenberg–Marquardt backpropagation algorithm as the training method due to its speed and accuracy in nonlinear data fitting.

To determine the optimal architecture, various network structures were evaluated by varying the number of neurons in the hidden layer. The best-performing configurations were selected based on the lowest prediction error and highest correlation between predicted and experimental data. The tangent sigmoid transfer function was used in both the hidden and output layers to introduce nonlinearity into the model.

The dataset used for ANN modelling was randomly divided into three subsets to ensure a robust and unbiased evaluation of the model’s predictive performance: 60% for training, 20% for validation, and 20% for testing. The training set exposed the network to sufficient data to learn the underlying patterns and relationships. The validation dataset was applied during training to monitor generalization, guide hyperparameter tuning, and enable early stopping to prevent overfitting. The testing set was reserved for final evaluation to assess the model’s ability to predict unseen data, confirming its real-world reliability.

## 3. Results and Discussion

### 3.1. Development and Validation

The RSM experimental design results highlighting the effects of process parameters on AFC 40 and AFC 80 membranes are presented in Table 2. The significant variation in responses across different experimental runs indicates that the independent variables substantially affected the rejection efficiency. A custom design approach was employed to study the combined effects of pressure, feed concentration, and flow rate on the pharmaceutical rejection process.

#### 3.1.1. ANOVA for Reduced Quadratic Model

Table 3 and Table 4 present the summary of the analyses of variance (ANOVAs), which were used to evaluate the significance of the quadratic model for caffeine and paracetamol, respectively. The quadratic model’s significance and independent terms were evaluated using Fisher’s F-test. This was calculated as the ratio of the model’s mean square to the residual mean square [33]. Each model term was examined for significance in terms of F-values and *p*-values. A high F-value is desired, and a *p*-value less than 0.05 shows the significance of the model; hence, it has an interaction effect on the response.

Table 3 displays the ANOVA regression model for caffeine rejection using the AFC 40 membrane. The model was significant, with an F-value of 282.59, and a *p*-value less than 0.0001 was obtained. The *p*-values for the process variables were less than 0.05, indicating that each model term had an interactive influence on the caffeine rejection.

Table 4 shows the ANOVA results for the reduced quadratic model of paracetamol rejection using the AFC 80 membrane. The model terms demonstrated lower *p*-values (*p* < 0.05) and higher F-values, indicating strong predictive capability for paracetamol rejection. Generally, very low *p*-values illustrate the significance of linear, quadratic, and interaction terms, establishing model validity for response prediction [34].

Both reduced quadratic models shown in Table 3 and Table 4 demonstrated excellent predictive capability, as confirmed by near-unity R^2^ values significantly exceeding the 0.90 threshold for robust fit in membrane separation studies [35]. For caffeine rejection, the model achieved an R^2^ of 0.9867 (adjusted R^2^ = 0.9832), explaining >98% of response variability. Similarly, paracetamol rejection was characterized by R^2^ = 0.9815 (adjusted R^2^ = 0.9723). The close agreement (<0.02 difference) between R^2^ and adjusted R^2^ for both models indicate minimal overfitting despite model complexity. These metrics collectively confirm exceptional alignment between experimental data and model predictions.

#### 3.1.2. Regression Equation

A quadratic polynomial was established to identify the relationship between the Y_i_ response and the various factors. The pharmaceutical rejection regression equations generated regarding the actual factors for AFC 40 and AFC 80 are Equations (4) and (5), respectively. Insignificant factors with *p*-values greater than 0.05 were removed from the initial model, and the reduced quadratic regression models in terms of actual factors were developed as follows:

Y_RSM (AFC 40)_ = +65.72100 − 0.636464X_1_ + 3.70135X_3_ + 0.095043X_1_X_3_ − 0.015017X_1_^2^ − 0.191160X_3_^2^ (4)

YRSM (AFC 80) = +81.35275 + 0.845238X_1_ + 0.231950X_2_ − 0.006345X_1_X_2_ − 0.012509X_1_^2^ (5)

where X_1_ is the pressure, X_2_ is the feed concentration, and X_3_ is the feed flow rate. As shown in Equation (4), the feed concentration was eliminated from the regression model, whereas pressure and feed flow rate were introduced as significant input parameters. For Equation (5), the feed flow rate has no significant effect on paracetamol, while pressure and feed concentration were introduced as significant input parameters.

#### 3.1.3. Effects of Transmembrane Pressure, Feed Concentration, and Flow Rate on Caffeine and Paracetamol Rejection Using RSM Plots

**Figure 4:**
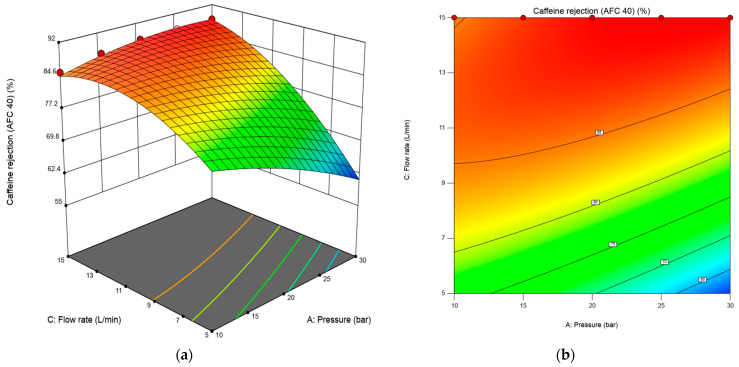
The 3D response surface (**a**) and 2D contour plot (**b**) for the interaction effect of flow rate and transmembrane pressure on the response for the AFC 40 membrane.

**Figure 5:**
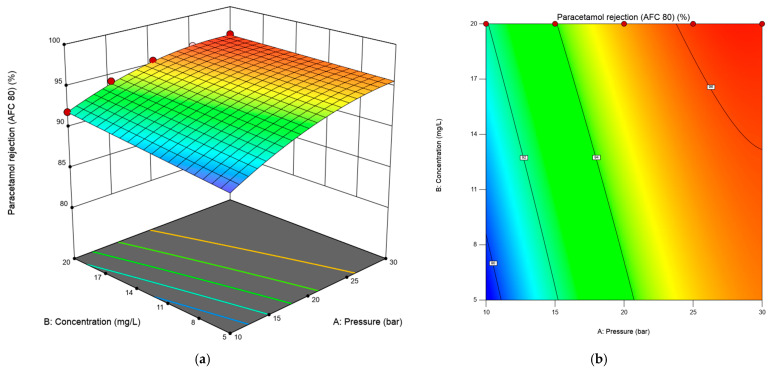
The 3D response surface (**a**) and 2D contour plot (**b**) for the interaction effect of concentration and transmembrane pressure on the response for the AFC 80 membrane.

Three-dimensional response surface plots and contour line maps of caffeine and paracetamol rejection rate, shown in Figure 4 and Figure 5, were obtained from the Design-Expert software to visualize the predicted models in RSM. The three-dimensional response surface plot in Figure 4a shows the relationship between two independent variables (transmembrane pressure and flow rate) and the response (caffeine rejection percentage). In contrast, the third variable (concentration) was constant at 10 mg/L. The corresponding 2D contour plot (bottom plane) displays lines of constant response, enabling identification of optimal operating zones. AFC 40 significantly affects pressure and flow rate in caffeine rejection. However, the effect of concentration was less critical. The 3D response surface in Figure 4a reveals a nonlinear interaction between pressure and flow rate in determining caffeine rejection by the AFC40 membrane. Below 10 L/min, increasing pressure from 10 to 30 bar resulted in a decline in caffeine rejection, possibly due to concentration polarization at the membrane surface, which can reduce effective rejection performance. In contrast, caffeine rejection increased steadily with pressure at higher flow rates, reaching up to 88.39%. This highlights the importance of optimizing both operating parameters simultaneously, as their interaction significantly influences separation efficiency.

The three-dimensional response surface and contour plot for paracetamol rejection versus concentration and transmembrane pressure are depicted in Figure 5a,b at a constant flow rate of 10 L/min. The plots were utilized to explain the process parameters’ combined effects on the percentage of paracetamol rejected by the AFC 80 membrane. The 3D response surface analysis demonstrated that paracetamol rejection is maximized (96.28%) at 30 bar and 20 mg/L, with both pressure and concentration exhibiting positive effects. The observed increase in paracetamol rejection as the feed concentration increases contradicts conventional membrane fouling behaviour, where higher solute concentrations typically reduce rejection due to fouling or concentration polarization [36,37,38].

This anomaly aligns with findings by Jorge Garcia-Ivars et al. [39], who observed similar concentration-enhanced rejection of paracetamol across three NF membranes. They proposed that at higher concentrations, paracetamol forms dynamic, loosely bound aggregates near the membrane surface. This secondary layer may enhance steric selectivity by blocking smaller solutes while allowing solvent to permeate through the membrane.

While these observations are compelling, the present study did not include targeted experiments or simulation-based approaches like Computational Fluid Dynamics modelling or membrane surface characterization to validate this mechanism. Therefore, the exact cause of the improved rejection remains speculative. Possible factors could include surface aggregation effects, specific solute–membrane interactions, or transient pore blockage, which merit further investigation. We propose that future studies incorporate such techniques to confirm whether the observed trend is due to aggregation-induced selectivity or other physicochemical phenomena. This will help clarify whether this behaviour is a membrane-specific effect or a generalizable feature across different NF systems treating paracetamol-laden wastewater.

### 3.2. Predictive Modelling Using ANN

The ANN architecture comprised interconnected layers of neurons that function like the human neural system [40], providing a robust framework for optimizing membrane processes. This data-driven framework enabled robust optimization of membrane performance beyond traditional RSM. The ANN was trained with the Levenberg–Marquardt backpropagation algorithm, one of the multilayer perceptron (MLP) networks used for error minimization. The Neural Network Toolbox V4.0 of MATLAB mathematical software was used to develop the ANN model.

The ANN was successfully developed using the experimental dataset presented in Table 2. The input variables for the neural network were identical to the factors considered in the RSM approach, namely, pressure, feed concentration, and feed flow rate. Similarly, the pharmaceutical rejection percentage was considered as the response variable for ANN modelling.

For the prediction of caffeine rejection, the optimal ANN architecture consisted of three input nodes, ten hidden neurons, and one output node, employing a tangent sigmoid transfer function in both the hidden and output layers. The Levenberg–Marquardt training algorithm provided the best prediction performance. In the case of paracetamol rejection, the most effective ANN configuration was a 3-11-1 structure, corresponding to three input parameters, eleven neurons in the hidden layer, and one output node.

#### Training, Testing, and Validation of ANN Model

The experimental data in Table 2 were partitioned into training, validation, and testing subsets to prevent overtraining and overparameterization. Training is the manipulation of input weights. The ANN was trained by iteratively adjusting input weights, which were initially randomized, to minimize the error between the predicted and actual rejection values [41]. The input vectors included pressure, flow rate, and feed concentration, while the output vector represented rejection efficiency. Once the ANN has been trained and tested with the appropriate weights, it can be used to predict the output [22].

**Figure 6:**
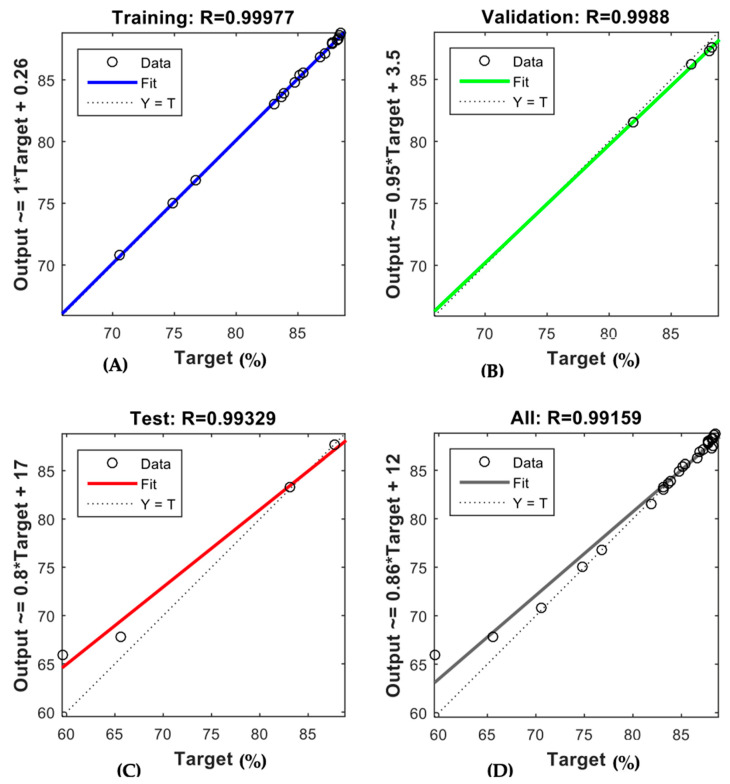
ANN regression plot for (**A**) training, (**B**) validation, (**C**) testing, (**D**) overall datasets of AFC 40.

**Figure 7:**
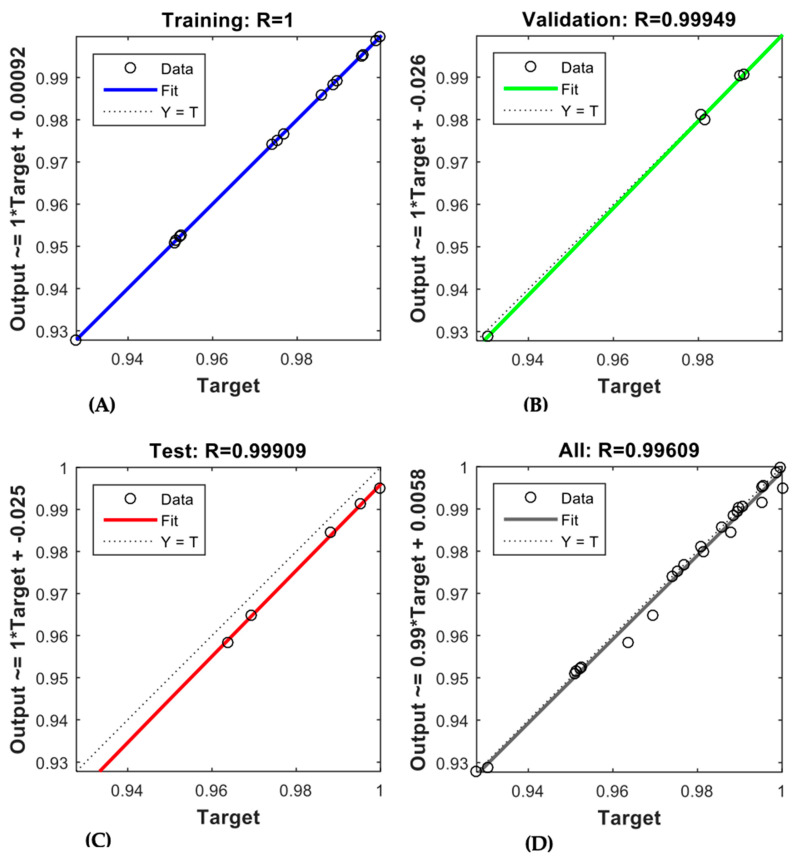
ANN regression plot for (**A**) training, (**B**) validation, (**C**) testing, (**D**) overall datasets of AFC 80.

The trained ANN demonstrated robust predictive capability for both membranes, as depicted in Figure 6 and Figure 7. The regression coefficients (R-values) obtained for the AFC 40 membrane were 0.99977, 0.9988, 0.99329, and 0.99159 for the training, validation, testing, and overall data, respectively. In comparison, the R-values for the AFC 80 membrane were even higher, recorded at 1, 0.99949, 0.99909, and 0.99609, respectively. These results confirm the model’s robustness in simulating the rejection behaviour of pharmaceutical compounds under varying operational conditions. Notably, the high R-values for the validation and testing phases show that the model performs well even with new data. This addresses a key gap in membrane modelling, where most traditional empirical models often struggle to predict membrane performance accurately when conditions change.

### 3.3. Comparative Study of RSM and ANN Models

RSM and ANNs are powerful modelling tools that address linear and nonlinear multivariate regression problems in membrane filtration studies [42]. The experimental, predicted, and residual values for pharmaceutical rejection by the AFC 40 and AFC 80 membranes are summarized in Table 2. While both models demonstrated reasonable accuracy in predicting rejection efficiency, a closer examination of residuals showing the differences between the experimental and predicted values revealed that the ANN consistently outperformed RSM, exhibiting significantly smaller deviations.

**Figure 8:**
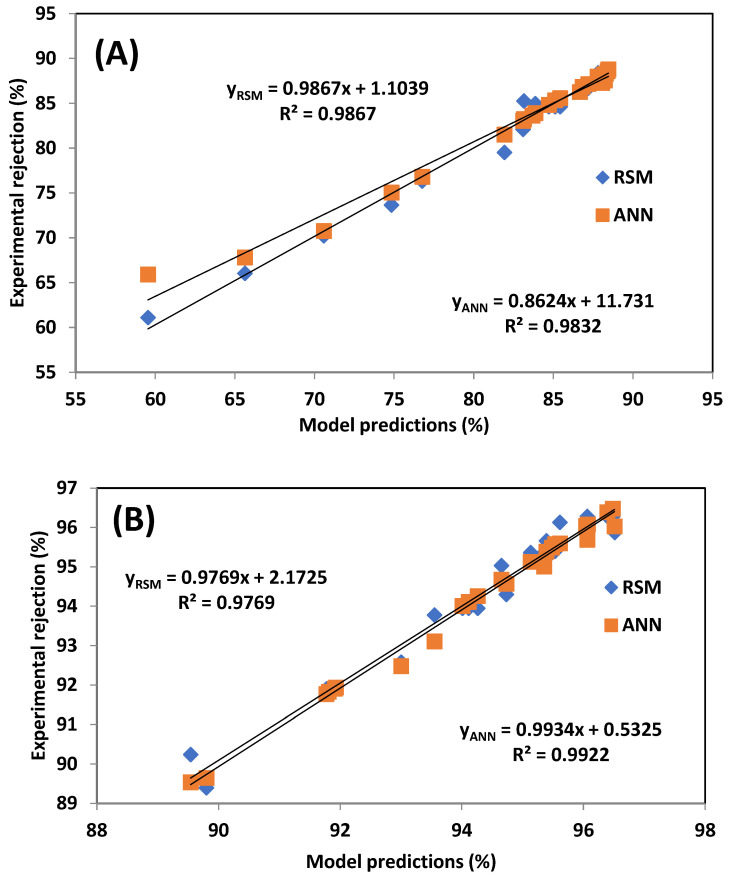
Comparative plots of experimental data with (**A**) RSM and ANN for caffeine rejection, (**B**) RSM and ANN for paracetamol rejection.

Visual validation of model performance is provided in Figure 8, which compares experimental results with RSM and ANN predictions. The plots confirm a strong correlation for both models, though ANN’s predictions align more tightly with the experimental data.

However, additional statistical analyses were used to further compare the models’ predicted pharmaceutical rejection values with the experimental efficiency and rank the models, as shown in Table 5. To evaluate the predictive accuracy of the RSM and ANN models for caffeine (AFC 40) and paracetamol (AFC 80) rejection, several statistical error functions were applied, including the Coefficient of Determination (R^2^), Root Mean Square Error (RMSE), Marquardt’s Percentage Squared Error Deviation (MPSED), Hybrid fractional error function (HYBRID), and Average Absolute Deviation (AAD). These error functions are defined by Equations (6)–(10), respectively:

(6) $$R^{2}=1-\frac{\sum_{i=1}^{n}\;{\left(Y_{i,p}-Y_{1,e}\right)}^{2}}{\sum_{i=1}^{n}\;{\left(Y_{i,p}-Y_{e,\mathrm{ave}}\right)}^{2}}$$

(7) $$\mathrm{RMSE}=\sqrt{\frac{\sum_{i=1}^{n}\;{\left(Y_{i,e}-Y_{i,p}\right)}^{2}}{n}}$$

(8) $$\mathrm{MPSED},\;\%=\sqrt{\frac{\sum_{i=1}^{n}{\left[\frac{Y_{i,e}-Y_{i,p}}{Y_{i,e}}\right]}^{2}}{n-P}}\times 100$$

(9) $$\mathrm{HYBRID},\;\%=\frac{1}{n-P}\sum_{i=1}^{n}\;\left[\frac{{\left(Y_{i,e}-Y_{i,p}\right)}^{2}}{Y_{i,e}}\right]\times 100$$

(10) $$\mathrm{AAD}=\frac{100}{n}\sum_{i=1}^{n}\;\left(\left|\frac{Y_{i,e}-Y_{i,p}}{Y_{i,e}}\right|\right)$$

where n is the number of experimental runs; Y_i,e_ is the experimental value of the ith experiment; Y_i,p_ is a model prediction; and P is the number of model parameters.

The ANN model demonstrated superior predictive capability, particularly in Table 5, which indicates that the ANN model generally provides better predictive accuracy and reliability than the RSM model, particularly in simulating paracetamol rejection using the AFC 80 membrane. In most cases, the ANN model yielded higher R^2^ values and lower error metrics, indicating stronger agreement between predicted and experimental values. Although the RSM model achieved slightly lower RMSE and HYBRID values for the AFC 40 membrane, the overall performance of the ANN, evidenced by its lower errors across a greater number of metrics and consistently higher R^2^ values, confirms its superior predictive capability. These findings are consistent with previous studies by Onu et al. [33], Addar et al. [26], and Ohale et al. [43], who also reported that ANNs outperformed RSM in modelling complex nonlinear processes.

While the controlled environment enabled precise parameter evaluation, real pharmaceutical wastewater introduces additional complexities due to the presence of dissolved salts, organic matter, and emerging contaminants. These additional constituents could alter membrane rejection behaviour through competitive solute interactions, fouling, or changes in electrostatic forces. Therefore, future studies are encouraged to validate the predictive power of RSM and ANN models using actual wastewater matrices to assess their robustness under more realistic treatment conditions.

## 4. Conclusions

A comparative study was conducted using RSM and ANN models to predict caffeine and paracetamol rejection by AFC 40 and AFC 80 NF membranes. The models incorporated three key operational variables: feed pressure, concentration, and flow rate.

Optimization using RSM enabled the identification of optimum operating conditions for both membranes. For the AFC 40 membrane, pressure and flow rate significantly influenced caffeine rejection, while feed concentration had a negligible impact. In contrast, for the AFC 80 membrane, pressure and feed concentration were the dominant variables influencing paracetamol rejection, with flow rate playing a minimal role. Both the RSM and ANN models demonstrated strong predictive capabilities in modelling pharmaceutical rejection.

The performance of both models was validated using statistical metrics, including R^2^, RMSE, MPSED, HYBRID, and AAD. For AFC 40, the R^2^ values were 0.9867 and 0.9832 for RSM and the ANN, respectively, while for AFC 80, they were 0.9769 (RSM) and 0.9922 (ANN). Although RSM yielded slightly lower RMSE and HYBRID values for AFC 40, the ANN model consistently produced lower error metrics across more parameters and achieved higher R^2^ values for both membranes.

The comparative analysis confirmed that the ANN model offered superior predictive accuracy and generalizability compared to the RSM model, particularly in capturing complex nonlinear relationships in membrane-based pharmaceutical rejection processes.

For further research, we recommend k-fold cross-validation and testing the predictive stability of the ANN model beyond the experimental dataset, especially by using independent datasets to assess its generalization across various industrial conditions.

## Figures and Tables

**Table 1:** Factors and levels of custom experimental design for membrane separation.

Factor or Independent Variable	Level 1	Level 2	Level 3	Level 4	Level 5
X_1_ (pressure, bar)	10	15	20	25	30
X_2_ (feed concentration, mg/L)	5	10	20	-	-
X_3_ (feed flow rate, L/min)	5	10	15	-	-

**Table 2:** Experimental and predicted responses of operating parameters.

Run	Independent Variables			Caffeine Rejection (AFC 40)			Paracetamol Rejection (AFC 80)			Model Residuals			
										(AFC 40)		(AFC 80)	
	X_1_ [bar]	X_2_ [mg/L]	X_3_ [L/min]	Y_Exp._ [%]	Y_RSM_ [%]	Y_ANN_ [%]	Y_Exp._ [%]	Y_RSM_ [%]	Y_ANN_ [%]	RSM [−]	ANN [−]	RSM [−]	ANN [−]
1	10	20	15	84.71	84.62	84.83	91.91	91.92	91.91	0.0897	−0.1195	−0.0143	0.0017
2	15	20	15	86.65	86.69	86.26	94.01	93.95	94.01	−0.0390	0.3919	0.0576	−0.0018
3	20	20	15	87.77	88.01	87.73	95.35	95.36	95.01	−0.2369	0.0412	−0.0051	0.3413
4	25	20	15	88.16	88.57	88.21	96.04	96.13	96.04	−0.4139	−0.0512	−0.0923	−0.0025
5	30	20	15	88.29	88.39	88.29	96.48	96.28	96.48	−0.1000	0.0031	0.1958	−0.0005
6	10	10	15	85.42	84.62	85.57	89.54	90.24	89.54	0.7997	−0.1503	−0.6993	−0.004
7	15	10	15	87.19	86.69	87.15	93.00	92.58	92.48	0.5010	0.0358	0.4154	0.5196
8	20	10	15	88.06	88.01	87.28	94.73	94.30	94.57	0.0531	0.7786	0.4254	0.1625
9	25	10	15	88.23	88.57	87.54	95.53	95.40	95.58	−0.3439	0.6924	0.1309	−0.0504
10	30	10	15	88.45	88.39	88.80	96.51	95.87	96.03	0.0600	−0.3528	0.6418	0.4831
11	10	5	15	85.11	84.62	85.34	89.80	89.40	89.65	0.4897	−0.2261	0.4032	0.1533
12	15	5	15	86.83	86.69	86.85	91.77	91.90	91.77	0.1410	−0.0179	−0.1308	0.0007
13	20	5	15	87.77	88.01	87.98	93.55	93.78	93.11	−0.2369	−0.2084	−0.2293	0.4361
14	25	5	15	88.40	88.57	88.59	94.65	95.03	94.68	−0.1739	−0.185	−0.3825	−0.0326
15	30	5	15	87.80	88.39	87.91	95.39	95.66	95.39	−0.5900	−0.111	−0.2702	0.0018
16	10	20	10	83.15	85.26	83.25	91.93	91.92	91.94	−2.11	−0.1032	0.0057	−0.0122
17	15	20	10	83.86	84.95	83.92	94.26	93.95	94.26	−1.09	−0.0642	0.3076	−0.0047
18	20	20	10	83.68	83.89	83.63	95.48	95.36	95.49	−0.2109	0.0502	0.1249	−0.0056
19	25	20	10	83.09	82.08	83.05	96.07	96.13	96.08	1.01	0.0375	−0.0623	−0.0057
20	30	20	10	81.92	79.52	81.51	96.39	96.28	96.39	2.40	0.4122	0.1058	0.0003
21	10	20	5	76.76	76.33	76.82	91.81	91.92	91.83	0.4255	−0.0613	−0.1143	−0.0205
22	15	20	5	74.83	73.65	75.05	94.11	93.95	94.11	1.18	−0.2214	0.1576	−0.0002
23	20	20	5	70.58	70.22	70.75	95.13	95.36	95.13	0.3631	−0.1731	−0.2251	−0.0012
24	25	20	5	65.62	66.03	67.81	95.61	96.13	95.60	−0.4117	−2.1909	−0.5223	0.0113
25	30	20	5	59.54	61.10	65.91	96.06	96.28	95.69	−1.56	−6.3702	−0.2242	0.3688

**Table 3:** ANOVA regression model (reduced quadratic) for caffeine rejection (AFC 40).

Source	Sum of Squares	df	Mean Square	F-Value	*p*-Value	
Model	1389.46	5	277.89	282.59	<0.0001	significant
A-Pressure [X_1_]	82.21	1	82.21	83.60	<0.0001	
C-Flow rate [X_3_]	1186.82	1	1186.82	1206.90	<0.0001	
AC	180.66	1	180.66	183.72	<0.0001	
A^2^	9.87	1	9.87	10.03	0.0051	
C^2^	85.65	1	85.65	87.10	<0.0001	
Residual	18.68	19	0.9834		<0.0001	
Cor Total	1408.14	24				
R^2^	0.9867					
Adjusted R^2^	0.9832					
Predicted R^2^	0.9645					
Adequate Precision	58.5621					

**Table 4:** ANOVA regression model (reduced quadratic) for paracetamol rejection (AFC 80).

Source	Sum of Squares	df	Mean Square	F-Value	*p*-Value	
Model	97.84	4	24.46	211.73	<0.0001	significant
A-Pressure [X_1_]	76.25	1	76.25	660.04	<0.0001	
B-Concentration [X_2_]	11.04	1	11.04	95.52	<0.0001	
AB	2.01	1	2.01	17.42	0.0005	
A^2^	6.85	1	6.85	59.25	<0.0001	
Residual	2.31	20	0.1155			
Cor Total	100.15	24				
R^2^	0.9769					
Adjusted R^2^	0.9723					
Predicted R^2^	0.9590					
Adequate Precision	45.3104					

**Table 5:** Statistical comparison of RSM and ANN models.

Error Function	AFC 40 Membrane		AFC 80 Membrane	
	RSM	ANN	RSM	ANN
R^2^	0.9867	0.9832	0.9769	0.9922
RMSE	0.8654	1.3700	0.3041	0.1999
MPSED (%)	0.0125	0.0512	0.0011	0.0004
HYBRID (%)	1.0893	3.5254	0.1124	0.0480
AAD (%)	0.7610	0.7720	0.2534	0.1101

## Data Availability

The original contributions presented in this study are included in the article. Further inquiries can be directed to the corresponding author.
